# Should We Assess Pituitary Function in Children After a Mild Traumatic Brain Injury? A Prospective Study

**DOI:** 10.3389/fendo.2019.00149

**Published:** 2019-03-19

**Authors:** Claire Briet, Karine Braun, Michel Lefranc, Patrick Toussaint, Bernard Boudailliez, Hélène Bony

**Affiliations:** ^1^Department of Pediatrics, University Medical Center, Amiens, France; ^2^Department of Endocrinology, Diabetology and Nutrition, Institut MITOVASC, INSERM U1083, Angers University, University Medical Center, Angers, France; ^3^Department of Neurosurgery, University Medical Center, Amiens, France; ^4^Department of Medicine, University of Picardie Jules Verne, Amiens, France

**Keywords:** traumatic brain injury, pituitary function, pediatric, hypopituitarism, growth hormone deficiency

## Abstract

**Objective:** The aim of this study was to evaluate the frequency of hypopituitarism following TBI in a cohort of children who had been hospitalized for mild TBI and to identify the predictive factors for this deficiency.

**Design:** A prospective study was conducted on children between 2 and 16 years of age who had been hospitalized for mild TBI according to the Glasgow Coma Scale between September 2009 and June 2013. Clinical parameters, basal pituitary hormone assessment at 0, 6, and 12 months, as well as a dynamic testing (insulin tolerance test) 12 months after TBI were performed.

**Results:** The study included 109 children, the median age was 8.5 years. Patients were examined 6 months (*n* = 99) and 12 months (*n* = 96) after TBI. Somatotropic deficiency (defined by a GH peak <20 mUI/l in two tests, an IGF-1 <-1SDS and a delta height <0SDS) were confirmed in 2 cases. One case of gonadotrophic deficiency occurred 1 year after TBI among 13 pubertal children. No cases of precocious puberty, 5 cases of low prolactin level, no cases of corticotropic insufficiency (cortisol peak <500 nmol/l) and no cases diabetes insipidus were recorded.

**Conclusion:** Pituitary insufficiency was present 1year after mild TBI in about 7% of children. Based on our results, we suggest testing children after mild TBI in case of clinical abnormalities. i.e., for GH axis, IGF-1, which should be assessed in children with a delta height <0 SDS, 6 to 12 months after TBI, and a dynamic GH testing (preferentially by an ITT) should be performed in case of IGF-1 <-1SDS, with a GH threshold at 20 mUI/L. However, if a systematic pituitary assessment is not required for mild TBI, physicians should monitor children 1 year after mild TBI with particular attention to growth and weight gain.

## Introduction

Traumatic brain injury (TBI) is a serious public health concern. Indeed, it is the principal cause of mortality and disability in children and young adults (1). Considering that the survival rate of children is higher than that of adults following severe TBI, studying TBI consequences is of interest (2). Indeed, mild TBI represents 80% of total TBI in children (1). In the last 15 years, retrospective studies have shown that pituitary deficiency is very common in children after TBI, but the frequency varies widely among studies (from 0.8 to 90% of children) (3–13).In these studies, GH deficiency (GHD) is the most common pituitary defect, but the diagnosis is based on biological data only. In the KIMS database (Pfizer International Metabolic Database reporting a follow up of patients under GH treatment), TBI accounted for 4% of GHD treated during childhood (14).

The prevalence of a pituitary deficiency after TBI in children cannot be reliably determined from the few published prospective studies, because of the small number of patients and partial information on the proportion of mild to moderate TBI (Table 1). The largest prospective study, including 87 children, focused on severe TBI (20). It interesting to note that almost all studies have concluded that TBI severity is not predictive of a pituitary deficiency (15, 20). Therefore, we focused our study on mild TBI.

**Table 1:** Prospective pediatric studies assessing pituitary deficiency 1 year after TBI.

**Source**	***N***	**GCS**	**Pit. def (%)**	**GHD (%)**	**GHD test**	**ACTH def (%)**	**ACTH axis test**
Einaudi et al. (5)	20	≤15	5	5	GHRH/O.S GH	0	Gluc. test
Kaulfers et al. (15)	31	≤12	29	5	Arg/O.S GH	0	Low dosage ACTH test
Norwood et al. (16)	32		25	16	Arg-gluc/O.S GH	19	Bas. cort
Aimaretti et al. (17)	23	≤15	30	30	GHRH–Arg	13	Bas. Cort/UFC
Jourdan et al. (18)	27	≤12		4	NA	0	Bas. cort
Ulutabanca et al. (19)	41	≤15	13	9	GHRH-arg	5	Bas. cort
Casano-Sancho et al. (4)	23	≤15+skull fracture	NA	39	Gluc/clonidine	13	Glucagon
Personnier et al. (20)	87	≤8	36	27	Gluc-betax/Arg-insulin	1	ACTH test
Krahulik et al. (21)	58	≤12	18	5	L dopa or ITT	3	Bas. cort /ITT

*N, number; GCS, Glasgow Coma Scale; Pit Def, Pituitary deficiency; def, deficiency; OS GH, overnight spontaneous GH; Arg, Arginine; gluc, glucagon; betax, Betaxolol; ITT, insulin tolerance test; Bas. Cort, basal cortisol 8 a.m*.

Here, we report the results of a large prospective study on 109 children (2–16 years) who had been hospitalized for mild TBI. The primary objective was to evaluate the prevalence of pituitary deficiencies in this cohort. The secondary objective was to determine predictive parameters of endocrine deficiency.

## Subjects and Methods

### Subjects

Inclusion criteria were age between 2 and 16 years old and admission to the Neurosurgery Unit of the University Hospital of Amiens (France) for medical supervision between September 2009 and November 2013 for TBI. The criteria for hospitalization in Neurosurgery after TBI in children in our hospital were: having experienced two episodes of vomiting, headaches, loss of consciousness, Glasgow Coma Scale <15, or neurological symptoms. The patients who met the criteria, agreed to participate in the clinical trial and came to the hospital for at least one evaluation (6 or 12 months after TBI) were included. Written and informed consent was obtained from the parents of all participants. Exclusion criteria were: children with a history of endocrine disease or TBI, or children who lived more than 150 km from our hospital.

The study protocol was approved by the French Ethics Committee (Comité de Protection des Personnes-2008-A00268-47), and the French General Direction of Health.

### Study Design

The Glasgow Coma Scale (GCS) was used to rank TBI as mild (GCS>12) (22, 23). Clinical parameters (height, weight, pubertal stage), were assessed at diagnosis and 6 and 12 months after TBI. Baseline pituitary function (IGF-I, FSH-LH, estradiol for girls, testosterone for boys, TSH-FT4, PRL, cortisol-ACTH, plasmatic osmolarity, and urinary osmolarity) was assessed at diagnosis and 6 and 12 months after TBI. Bone age was assessed at diagnosis and at 12 months. At 12 months, an insulin tolerance test (ITT) was performed for somatotropic and corticotropic axis assessment, given that it is considered the “gold standard” with fewer false positive results (24).

After overnight fasting, intravenous insulin (0.075 UI per kilogram for body weight below 15 kg, otherwise 0.1 units per kilogram) was administered to patients and samples were taken for blood glucose, cortisol and growth hormone (GH) measurement at 0, 15, 30, 45, 60, 90, and 120 min after injection.

In the event of a seizure at the time of TBI, an arginine test was performed for somatotropic evaluation, as well as a synacthen test (250 μg IV with cortisol dosage at −30, 30, and 60 min) for corticotropic evaluation.

Dynamic testing of the corticotropic and somatotropic axis was performed 12 months after TBI. Therefore, the prevalence of pituitary hormone deficiencies is reported only at 12 months.

### Hormonal Assessment

GH, IGF-1, FT4 were assessed by radioimmunoassay (CisBio International for GH and IGF-1, Beckman-Coulter for FT4) with a sensitivity of 0.03 mUI/ml, 2 ng/ml, and 0.5 pmol/l, respectively. Cortisol was assessed using a Beckman Access kit with a sensitivity of 1.1 nmol/l. ACTH, prolactin, LH and FSH were assessed with immunoradiometric assays (Beckman Coulter) with a sensitivity of 1.2 pg/ml, 0.5 ng/ml, 0.2 UI/ml, 0.2 UI/ml, respectively. Estradiol was assessed with E2—Coatria, CisBio International with a sensitivity of 4 pg/ml.

Inter/intra-assay coefficients of variation were 4.5%/1.7% for GH, 3.8%/3.8% for IGF-1, 7.5%/8.3% for FT4, 7.9%/6.7% for cortisol, 9.6%/9.1% for ACTH, 8%/2.8% for prolactin, 4.9%/3.9% for estradiol, 15%/14.8% for testosterone, 3.7%/6.7% for LH, and 6.3%/2.6% for FSH.

The pediatric normal values published in the literature were used to diagnose pituitary dysfunction:

– Diabetes insipidus was suspected in cases of nocturnal drinking combined with low morning urine osmolarity (<300 mmol/kg) (25, 26).
– Hypocortisolism was defined as a peak of cortisol <500 nmol/l with documented hypoglycemia (blood glucose <2.2 mmol/l (27–29).
– Thyrotropin insufficiency as FT4 level below the normal range (from <11.96 to 14.29 pmol/l according to age) and normal or low TSH level (normal value from 0.57–5.51 to 0.5–4.9 mUI/l according to age) (30, 31).
– Hypogonadotropic hypogonadism was diagnosed
  ◦ in pubertal girls: by secondary amenorrhea or alteration of menses regularity (from regular menses to irregular menses) after age of 13 and low estradiol levels with low FSH and LH levels (<1 and 0.4 mUI/l, respectively (32),
  ◦ in pubertal boys: with complete puberty (genital and pubertal stage 5) by erectile dysfunction (lack of spontaneous morning erection) after the age of 15 and a testosterone below 10.4 nmol/l (3 ng/ml) with low FSH (< 1.2 mUI/l) and LH (2.4 mUI/l) (32, 33).

Hypogonadotropic hypogonadism was not evaluable in prepubertal children, for these children, signs of precocious puberty were investigated: acceleration of height velocity, advanced bone age, and enlargement of mammary gland before 8 years of age for girls or increased testes volume (> 25 × 20 mm or 4 ml) before 9 years for boys (30, 34).

– The somatotropic axis was evaluated by an ITT with documented hypoglycemia (blood glucose <2.2 mmol/l) (35, 36). In the event of GH peak <20 mUI/l, a second test was performed (arginine test) within the following 3 months. The arginine test was performed with a 30-min IV perfusion of arginine 0.5 g/kg (maximum 25 g), with blood samples every 15 min from 30 to 90 min. IGF-1 was expressed in standard deviation for age, sex, and pubertal stage (SDS). We explored the consequences of 3 definitions of GHD according to various criteria:
  ◦ **Level 1**: *biochemical GHD* defined by two GH peaks <20 mUI/L in response to stimulation tests. Two stimulation tests are used because of the low specificity of dynamic testing (37). The cut off used for GHD treatment in children in France is 20 mUI/l (6.7 μg/l) which is approved in the literature (38, 39). However, if there is a consensus for GHD diagnosis in adults with a GH peak after ITT <3 μg/l (9 mUI/l) and 6 μg/l (18 mUI/) in the transition period, there is no consensus for GH cut-off after ITT in children (16, 19),
  ◦ **Level 2:** *biochemical GHD* + *low IGF-1*: two GH peaks <20 mUI/L in response to stimulation tests and IGF-1 <- 1 SDS, as recommended by the GH Research Society as a way of counterbalancing the low specificity of GH stimulation tests, especially in obese children and because most children with GHD have an IGF-1 below−1 SDS as described in the literature (40–43),
  ◦ **Level 3:** *biochemical GHD* + *low IGF-1* + *growth deficiency*: two GH peaks <20 mUI/L in response to stimulation tests, an IGF-1 <-1 SDS and a decrease in height velocity (delta height < 0 SDS). We used these stringent criteria for GHD diagnosis as a way of avoiding any false positive diagnoses of GHD and because GHD had a deficiency on growth in children.

We presented the results for the three levels of GHD mainly for the discussion of GHD frequency according to these levels and comparison with previous studies. However, for the diagnosis of GHD in our cohort, the most stringent criteria (GHD level 3) were adopted.

– Hyperprolactinemia was defined by PRL above 20 ng/ml, hypoprolactinemia was defined by a PRL below 3 ng/ml.
– Bone age was assessed through an X-ray of the left hand, finger and wrist and was calculated by the same pediatrician according to the Greulich and Pyle atlas at M0 and M12.

### TBI Severity

Mild TBI is defined by a CGS > 12 and was further characterized as complicated or uncomplicated mild TBI, according to the presence or absence of CT-Scan abnormalities such as a depressed skull fracture or other trauma-related intracranial abnormalities (e.g., hemorrhage, contusion, and edema) (44).

### Statistical Analysis

The sample size calculation required was one hundred and twenty patients, comparing those who have one or more pituitary deficiency to those without, to ensure a statistical power of 80% for an odd ratio of 1.5 to 2.5 (prevalence of one or more pituitary deficiencies at around 20% according to the literature review) with 10% lost to follow up.

Data were expressed as median, range and percentage of abnormal responses compared to normative cut-off levels.

Statistical analysis in various groups was performed using the Wilcoxon rank-sum test. The percentages were compared using the chi-squared test, with Fisher correction.

The predictive factor for developing a pituitary deficiency was calculated using logistic regression analysis including age, sex, initial presentation (GCS score, vomiting, scan abnormalities, loss of consciousness), pubertal status and BMI, adjusted with prealbumin levels as a marker of malnourishment which can modify GH response to stimulation tests and IGF-1 levels. We excluded children with previous TBI and endocrine disorders as potential confounders. In the same way, we excluded children under 2 years of age to avoid shaken baby syndrome.

A two-tailed *p* value < 0.05 was considered statistically significant. The statistical analysis was performed using GraphPad Prism version 4 software for Windows (GraphPad Software, San Diego, California, USA) and with Statistical Analysis System version 9.2 software for Windows for the logistic regression analysis (SAS Software, L9.2G, North Carolina, USA).

## Results

### Patients

Of the 139 children admitted to the Neurosurgery Unit between September 2009 and June 2013, 30 were excluded: 1 had a history of precocious puberty, 6 lived too far from the university hospital to participate in the follow-up, 14 parents declined to participate, and 9 children did not return for a 6-month and 12-month evaluation (Supplemental Figure 1).

The characteristics of the 109 patients included are summarized in Table 2. The median age was 8.5 years, and 73% were at pubertal stage 1 at M0. Ninety-Nine and Ninety-Six patients were evaluated at Six and Twelve months, respectively. The median height of the total group was 0.53 SDS (−1.9 to 3.2), median weight 0.03 SDS (−1.7 to 6.2) and median BMI −0.49 SDS (−2.8 to 10).

**Table 2:** Demographics, injury characteristics and imaging results of the 109 patients at M0 (102 CT scan realized).

**Description of the patient sample**		
*N*	109	
Age at TBI, years (median [range])	8.5	2–16.2
Gender M/F	73/36	
GCS median [range]	15	(13–15)
Mechanism (%) – Impact – Falls – Cycler – Motor vehicle	9% 55% 12% 23%	(10/109) (60/109) (13/109) (26/109)
Unconscious <1 min >1 min	29% 50% 50%	(30/109) (15/30) (15/30)
Seizure	1%	(1/109)
Intubation	0%	(0/109)
Overview of CT scan findings – Diffused swelling – Pneumocephalus – Meningeal hemorrhage – SDH – EDH – Skull fractures – Fracture + swelling – Normal No CT scan	7% 5% 4% 2% 15% 13% 4% 53% 3%	102 scan (7/102) (5/102) (4/102) (2/102) (15/102) (13/102) (4/102) (54/102) (3/109)

*SDH, subdural hematoma; EDH, extra dural hematoma*.

### Endocrine Evaluation

#### Somatotropic Axis

One year after TBI, 10 out of 96 children had GHD level 1 (two tests with GH peak <20 mUI/l). Among these 10 children, 3 had IGF-1 <-1 SDS and were labeled GHD level 2. Among them, 2 had delta height <0 SDS between M0 and M12 (GHD level 3). Between the two patients, one had a GH peak <10 mUI/l. No IGF-1 below−2 SDS was found in children with GHD regardless of the level. The median height of children with GHD was 0.53 SDS (−0.27 to 1.8) and the median BMI 0.28 SDS (−0.18 to 3.6). For the complete description of children with GHD level 1, 2, and 3 see Table 3.

**Table 3:** Auxological and hormonal data of patients with two stimulated GH peaks below 20 mUI/l (GHD level 1 in white), with two peaks below 20 mUI/l and IGF-1 <-1SDS (GHD level 2 in light gray) and with the previous criteria and delta height <0 (GHD level 3 dark gray).

***N***	**Age range (year)**	**CGS**	**CT scan**	**Height M0 (SDS)**	**Delta height M6/M12 (SDS)**	**Delta weight M6/M12 (SDS)**	**GH peak mUI/l (1)**	**GH peak mUI/l (2)**	**IGF-1 M0/M6/M12 (SDS)**	**Cort peak nmol/l (1/2)**
15	5–10	15	Swelling	2.15	0.08/0.24	−0.12/0.17	7.78	10.7	−1.11/−0.96/−0.89	N
34	5–10	13	Swelling	0.53	0.16/0.19	0.65/0.7	13.4	17.3	−1.85/−0.86/−0.96	N
61	5–10	15	No	2.3	−0.2/−0.03	0.43/1.1	15.7	13.1	−0.5/−0.12/1.06	N
79	5–10	15	No	0.53	NA/−0.1	NA/−0.54	4.8	17.9	−1.52/NA/−0.84	N
80	10–15	15	Normal	1.28	−0.14/−0.22	−0.17/−0.89	13.7	19	−0.47/−1.01/−0.13	N
12	5–10	13	Swelling	NA	NA	−0.46/−0.24	6.21	14.9	NA/−0.03/1.7	N
28	5–10	15	SDH	0.19	−0.27/−0.23	0.04/−0.23	14.8	10.7	−0.7/−0.22/−0.83	N
95	5–10	13	Fracture	−0.73	0.23/0.09	0.28/0.5	14.3	16.7	−1.64/−1.52/−1.62	N
43	5–10	15	Fracture	1.5	−0.11/−0.23	−0.6/−0.46	12.9	11,1	−1.64/−1.89/−1.98	463/507
63	5–10	15	Normal	−0.8	0.04/−0.24	0.15/−0.24	3.9	4.92	−1.93/−1.77/−1.78	N

*M, male; F, female; imp, impuberism; N, normal; SDH, subdural hematoma*.

By targeting children with a growth deficiency at M12 (with a delta height below 0 SDS), 63 children would have been selected. Among them 24 had an IGF-1 < −1SDS. Among the 24 children, 2 children had a GH peak after two stimulation tests < 20 mUI/l (GHD level 3). This strategy for screening children after TBI would have 100% sensibility and 34.4% specificity.

#### Corticotropic Axis

The ITT revealed 28 corticotropic deficiencies, according to the 500 nmol/l threshold, among the 96 children tested. An ACTH stimulation test was performed on 18 children within 3 months, none had a cortisol peak <500 nmol/l.

#### Thyrotropin, Axis

**Figure 1:**
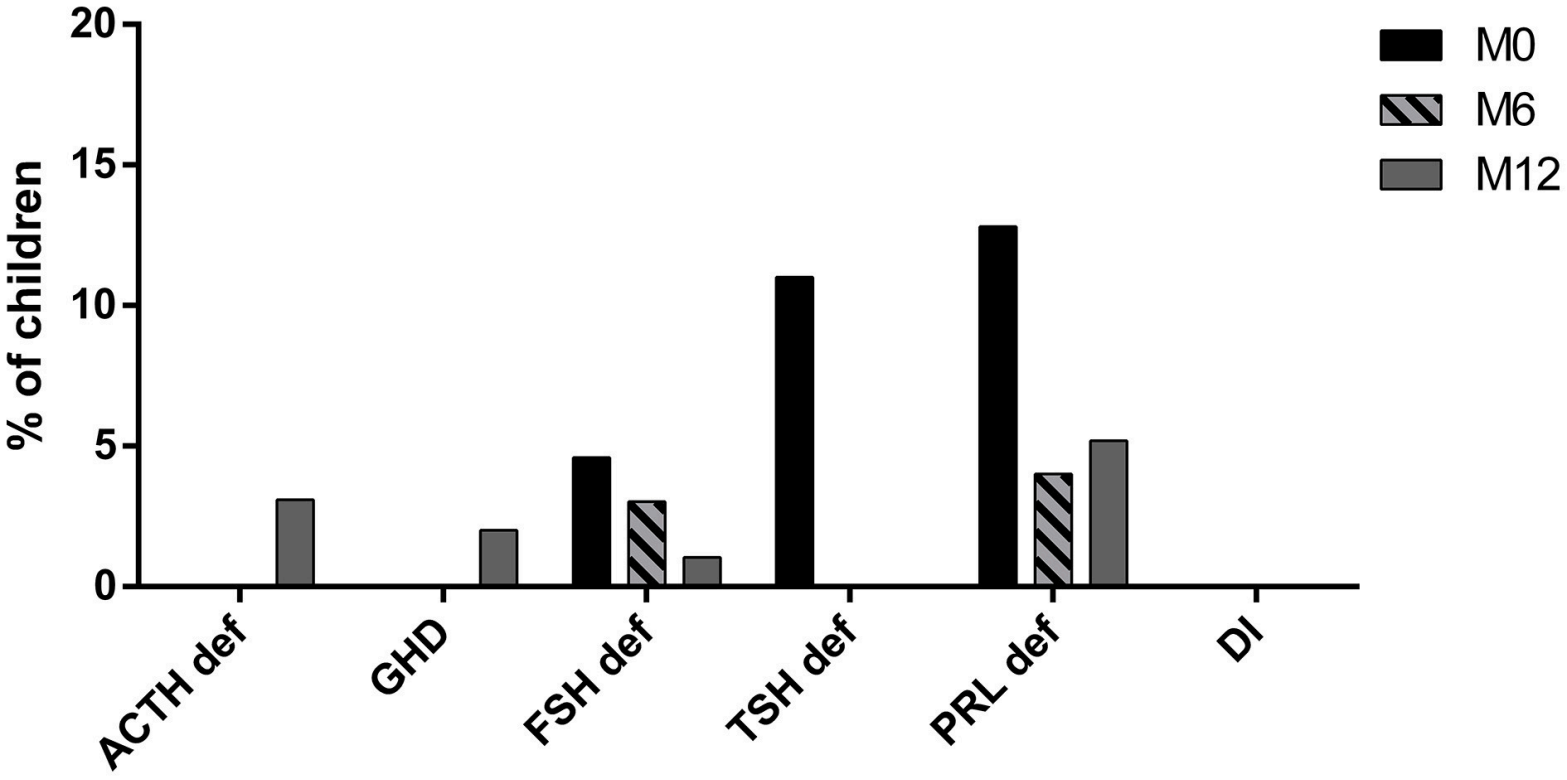
Pituitary evaluation at traumatism (M0), 6 months (M6) and 12 months (M12) after TBI. (PRL def, prolactin deficiency; TSH def, TSH deficiency; FSH def, gonadotropin deficiency; DI, diabetes insipidus; GHD, GH deficiency; ACTH def, ACTH deficiency).

Upon first investigation, immediately after TBI, there were 12 cases of thyrotropin deficiency among 109 patients; all of whom recovered at 6 months (Figure 1). A subclinical hypothyroidism (elevated TSH and normal FT3 and FT4) was found in one case at M0 and 2 cases at M12.

#### Gonadotropin, Prolactin Axis and Posterior Pituitary Evaluation

At M0, 14 of 109 children had a prolactin deficit (10 had recovered at 6 months and 1 new deficiency was diagnosed at 12 months). Out of 10 children who were pubertal at presentation, 5 had a gonadotropin deficiency (3 recovered at 6 months and 1 recovered at 12 months). At 12 months, one case of gonadotropin deficiency persisted among 13 late adolescents. There were no cases of precocious puberty among 62 prepubertal children. Morning urine osmolarity was low in one child who began drinking during the night, but the suspicion of DI was not confirmed by the total urinary volume (less than 50 ml/kg) (Figure 1).

To summarize the hormonal results, at 12 months, 7 out of the 96 children (7.2%) tested had one or more pituitary deficiencies except for the gonadotropic axis which was evaluated according to age. Comparison of the demographic, clinical, biochemical and radiological data in these patients highlighted no differences between patients with a normal pituitary function and patients with pituitary deficiencies in terms of age, gender, CT scan abnormalities, unconsciousness at presentation, weight gain, delta height gain, CGS.

### Influence of TBI Severity

**Figure 2:**
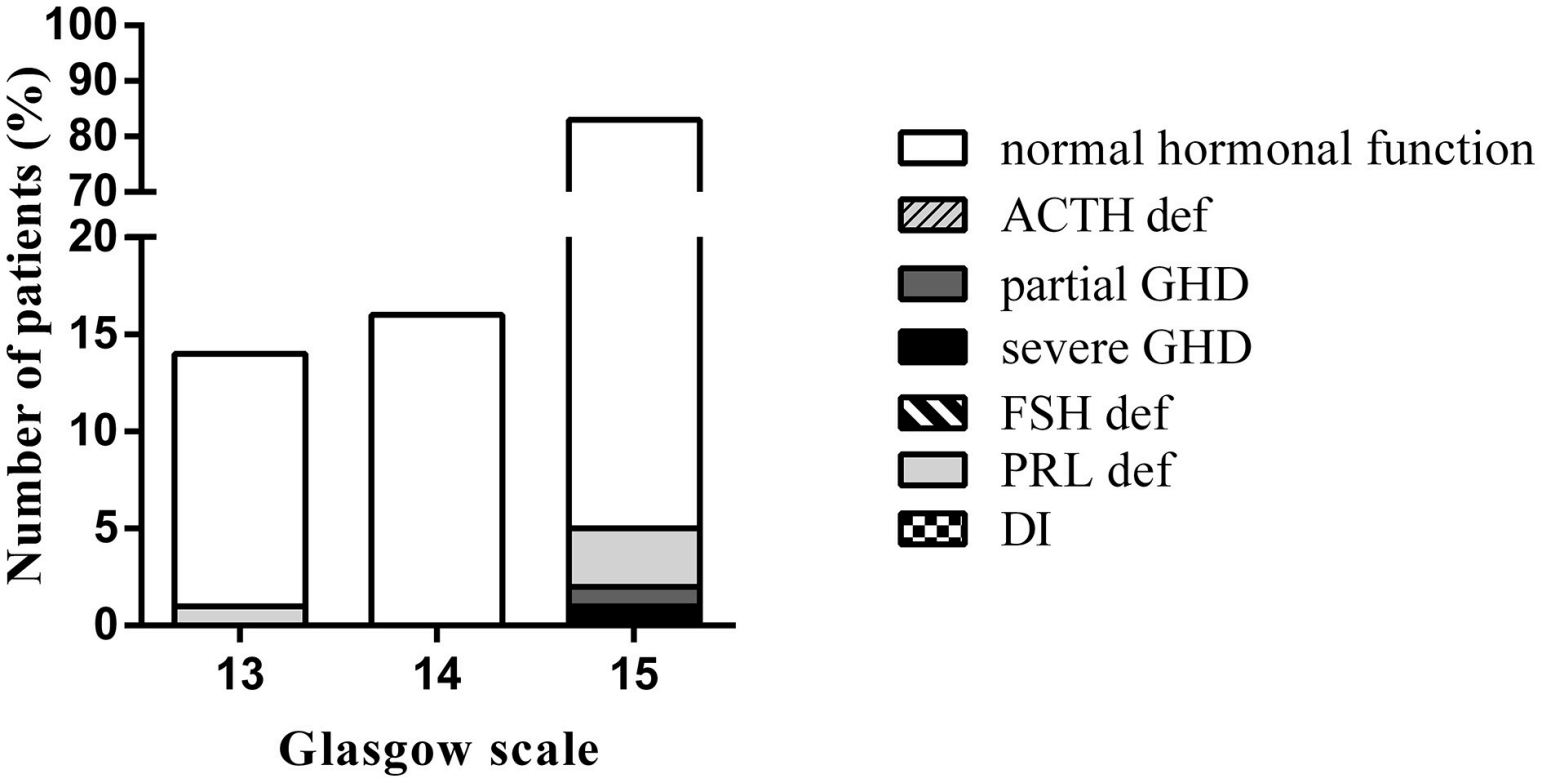
Comparison of pituitary function at 12 months, according to the Glasgow Coma Scale. (DI, diabetes insipidus; FSH def, gonadotropin deficiency; PRL def, prolactin deficiency; ACTH def, ACTH deficiency; GHD, Growth hormone deficiency).

In our study, pituitary deficiency occurred even in mild TBI with a CGS score at 15 (Figure 2).

**Figure 3:**
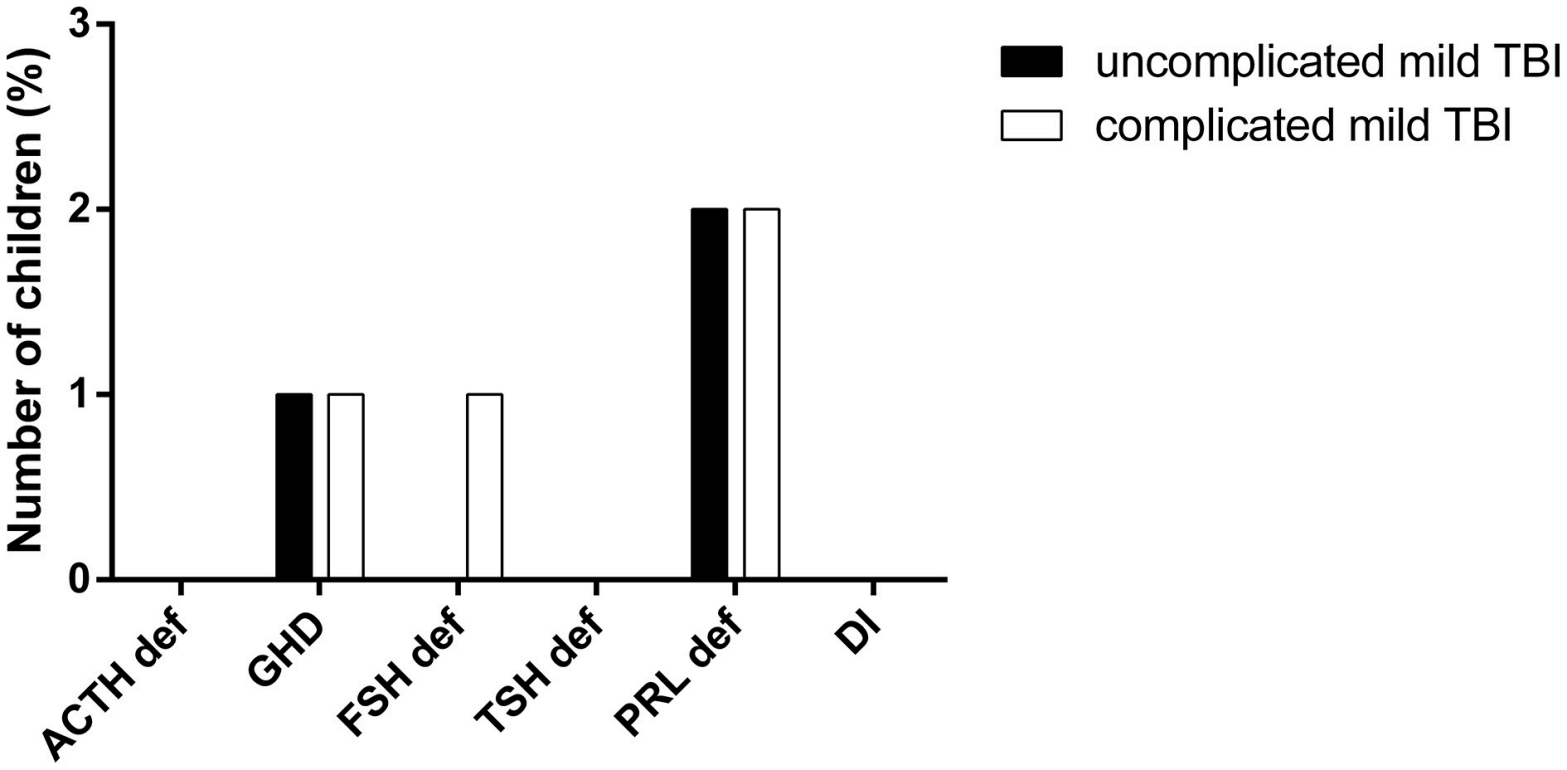
Comparison of hormonal deficiencies in complicated or uncomplicated mild TBI. (PRL def, prolactin deficiency; TSH def, TSH deficiency, FSH def, gonadotropin deficiency, DI, diabetes insipidus, GHD, GH deficiency, ACTH def, ACTH deficiency).

When splitting the cohort into two subgroups: complicated or uncomplicated mild TBI, according to the presence or absence of CT-Scan abnormalities, there were no differences between the two subgroups (Figure 3).

### Predictive Factors

Clinical and biological parameters at M0 (vomiting, GCS, scan abnormalities, loss of consciousness, pubertal status, BMI, age, hormonal level at TBI) were not predictive of pituitary insufficiency.

## Discussion

We prospectively assessed pituitary hormonal deficiency in a cohort of children and adolescents who had been hospitalized for mild TBI.

Twelve months after TBI, we found that 7.2% of patients suffered from pituitary deficiencies which is much less than previous prospective studies (Table 1) have recorded. These differences can be explained by the stimulation test used (ITT), which is more sensitive, and by the stringent criteria used to define pituitary deficiency (GHD level 3 and cortisol peak <500 nmol/l after two stimulation tests). We choose a cut-off for the GH peak of 20 mUI/l (6.7 μg/l) as recommended by the GH research society and as used in France for GH treatment (40). GHD, as assessed by a GH peak below 20 mUI/l following two consecutive stimulation tests, an IGF-1 <-1SDS and a delta height <0SDS (GHD level 3) was present in 2 children who were treated. In the literature, GHD is the most frequent pituitary deficiency, but the definition does not include the growth defect in most published research. Furthermore, the cut off values for GH peak and the stimulation tests were often different (45, 46).

A GHD level 1 or 2 with normal growth following TBI in children is common in the literature. Considering GHD when only a hormonal deficiency has an impact on growth, the percentage of children with a diagnosis of GHD falls from 10 to 2% in our study, and from 39 to 3.8%, 5 to 0% or 30 to 2% in the series reported by Casano Sancho et al., Kaulfers et al., Personnier et al., respectively (4, 15, 20). By combining a low level of IGF-1 and a GH peak below 20 mUI/l, as recommended by the GH Research Society (47), 3 children fulfilled the criteria for GHD in our study (48). Given that many children had a high BMI in our cohort, which leads to physiologically low GH peaks in response to stimulation, the addition of a low IGF-1 as a criterion of GHD could be a discriminant in this subpopulation. However, the discrepancy between two dynamic tests can reach 50% and children with normal growth can have a low GH peak following a GH stimulation test (from 44 to 61% for children Tanner 1 and 2, respectively), IGF-1 measurement has a high variability and a GHD with normal IGF-1 is possible (24, 37, 49). It is therefore difficult to define GHD after TBI: should it be determined by dynamic testing alone, or by the combination of dynamic testing, basal IGF-1 and/or with a decreased delta height? This is an important point as in adults, the lack of impaired growth as criterion for GHD, may partially explain the differences in GHD frequencies after TBI between children and adults (50).

Bellone et al. recommend the evaluation of height velocity and basal hormonal testing at 6 and 12 months after TBI and performing dynamic testing only on children with impaired height velocity (<25e percentile) (6). With regard to our results, we recommend monitoring height velocity at 0, 6, and 12 months after TBI, and measuring IGF-1 in children with a delta height <0 SDS 6 or 12 months after TBI. In case of IGF-1 <-1 SDS, a dynamic testing should be proposed, preferably an ITT in absence of contraindication with a threshold of GH at 20 mUI/l. With this strategy, 63 children would have been selected and tested for IGF-1. Among them 24 children with an IGF-1 < −1SDS would have been tested for GH secretion. Among the 24 children, 2 children with GHD would have been diagnosed.

In our study the incidence of cortisol deficiency was 29% with a threshold of 500 nmol/l, which was not confirmed following a second test. Three children had a second cortisol peak between 500 and 550 nmol/l. In the literature, the frequency of cortisol deficiencies after TBI is variable (0 to 19%), mainly because different stimulation tests and variable cut off's are used (summarized in Table 1).

In contrast to other studies, we did not observe any precocious or delayed puberty, probably due to the mean age of the cohort (with a majority of Tanner 1 children) and the short follow-up (5, 9, 15). A gonadotrophic deficiency was diagnosed in one girl with an impaired cycle, low estradiol and low FSH-LH. There were only 13 pubertal children at 12 months, it is therefore difficult to draw any conclusions on the frequency of abnormalities for this axis. A prolonged follow-up should provide further information.

In our study, pituitary deficiency is not correlated with a lower GCS at TBI, as in some prospective studies (15, 20). TBI severity is commonly evaluated with GCS, even if it is not the optimal score (51). Therefore, we tried to complement the GCS score for mild TBI by including the TDM result (complicated and uncomplicated mild TBI). However, this classification did not change the predictive value of TBI severity.

Various blood biomarkers of TBI severity were recently proposed and correlated with injury severity and outcome: Ubiquitin C-terminal Hydrolase-L1 and Glial Fibrillary Acidic Protein, Interleukin 10, neuron specific enolase, neurogranin, metallothionein, S100B, tau, estrone. These could help to assess TBI severity at diagnosis more effectively (52–57).

In this longitudinal study, we did not find any new deficit between 6 and 12 months except for prolactin deficiency which is difficult to define in prepubertal children. Nevertheless, further evaluation should be interesting in this cohort 3 years after TBI, especially regarding putative gonadotropic deficiencies, or precocious puberty.

The strength of this study is its large number of children, and its use of the insulin tolerance test which is the gold reference test and the most suitable test after TBI (3, 58). The absence of a control group (for ethical reasons), appears to be a limitation, but the tests used here have been largely accepted in the general pediatric population. This study sheds lights on several problems in endocrinology: the fragility of hormonal testing, the threshold in endocrinology, and the difficulties in diagnosing GHD in patients after TBI, particularly in adulthood where the growth parameter is lacking.

## Conclusion

This study calls the attention to endocrine dysfunction after TBI, even for mild TBI. In this study, we could stress the value of a delta height < 0 SDS is a warning, which should lead to an IGF-1 measurement, and dynamic testing in case of IGF <-1 SDS. The basic care of children should include the assessment of growth and weight with particular attention to the year following mild TBI.

## Data Availability

All datasets generated for this study are included in the manuscript and/or the supplementary files.

## Author Contributions

CB, KB, and ML contributed to the recruitment and follow up. CB, KB, PT, BB, and HB contributed to the redaction of the manuscript.

### Conflict of Interest Statement

The authors declare that the research was conducted in the absence of any commercial or financial relationships that could be construed as a potential conflict of interest.
