# Supplementary figures and images for: Should We Assess Pituitary Function in Children After a Mild Traumatic Brain Injury? A Prospective Study
Source: Front Endocrinol (Lausanne). 2019 Mar 19;10:149. doi: 10.3389/fendo.2019.00149 (PMC6433821; doi:10.3389/fendo.2019.00149)

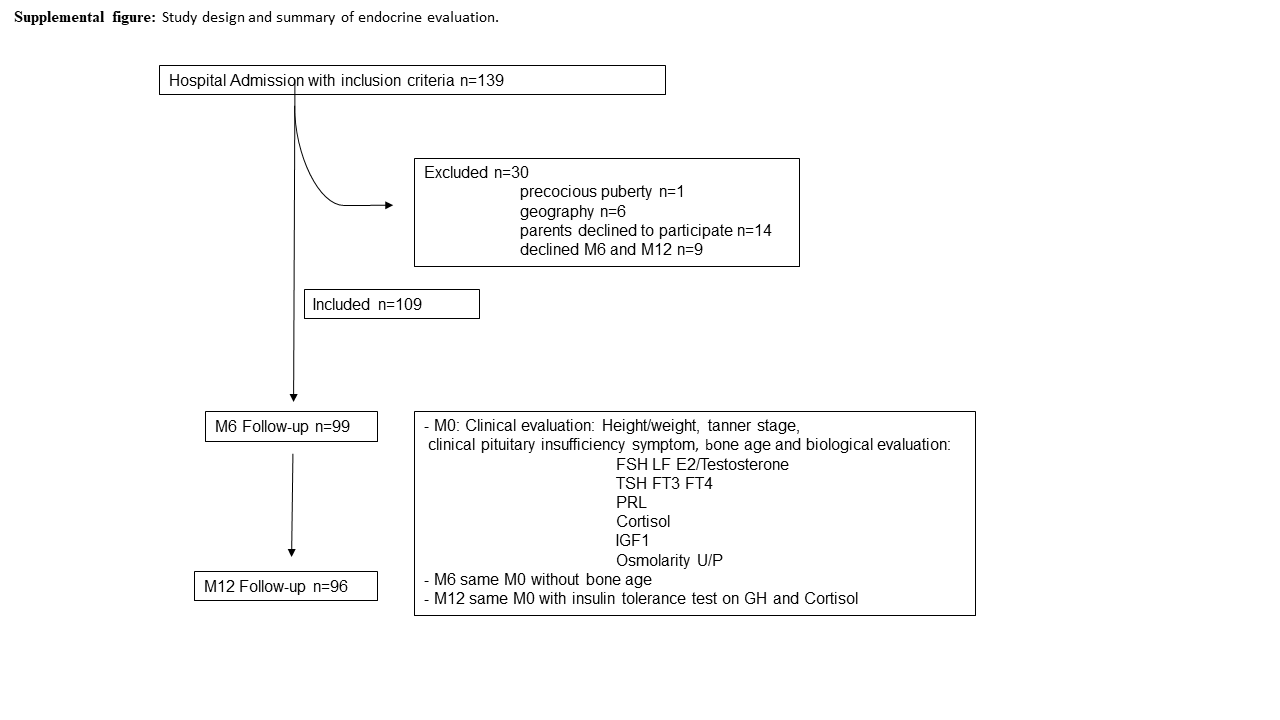

Supplement: Supplementary file 1 [file Image_1.TIF]
